# Enhanced performance of a novel anodic PdAu/VGCNF catalyst for electro-oxidation in a glycerol fuel cell

**DOI:** 10.1186/s11671-017-2360-x

**Published:** 2017-11-25

**Authors:** N. Yahya, S. K. Kamarudin, N. A. Karim, M. S. Masdar, K. S. Loh

**Affiliations:** 1 Fuel Cell Institute, Universiti Kebangsaan Malaysia, UKM, 43600 Bangi, Selangor Malaysia; 2 Malaysian Institute of Chemical and Bioengineering Technology, Universiti Kuala Lumpur, Melaka, Malaysia; 3 Department of Chemical and Process Engineering, Faculty of Engineering and Built Environment, Universiti Kebangsaan Malaysia, 43600 Bangi, Selangor Malaysia

**Keywords:** Glycerol fuel cell, Anodic catalyst, Palladium-gold alloy

## Abstract

This study presents a novel anodic PdAu/VGCNF catalyst for electro-oxidation in a glycerol fuel cell. The reaction conditions are critical issues affecting the glycerol electro-oxidation performance. This study presents the effects of catalyst loading, temperature, and electrolyte concentration. The glycerol oxidation performance of the PdAu/VGCNF catalyst on the anode side is tested via cyclic voltammetry with a 3 mm^2^ active area. The morphology and physical properties of the catalyst are examined using X-ray diffraction (XRD), field emission scanning electron microscopy (SEM) and energy dispersive X-ray (EDX) spectroscopy. Then, optimization is carried out using the response surface method with central composite experimental design. The current density is experimentally obtained as a response variable from a set of experimental laboratory tests. The catalyst loading, temperature, and NaOH concentration are taken as independent parameters, which were evaluated previously in the screening experiments. The highest current density of 158.34 mAcm^−2^ is obtained under the optimal conditions of 3.0 M NaOH concentration, 60 °C temperature and 12 wt.% catalyst loading. These results prove that PdAu-VGCNF is a potential anodic catalyst for glycerol fuel cells.

## Background

Conventional sources of energy, such as fossil fuel, are limited and will someday be depleted. Though the consumption of fossil fuels remains a necessity, the combustible materials we use as fuel cannot be replaced quickly enough to meet future energy demands [1, 2]. Fuel cell is a promising renewable energy technology that combines hydrogen and oxygen to produce electricity, heat and water. Previously, hydrogen has been used as the basic fuel for fuel cells. Unfortunately, the difficult handling and storage of hydrogen require further research to replace hydrogen with liquid fuel as an energy carrier and to deliver hydrogen to a fuel cell [3].

In early research, methanol was the most common fuel used in fuel cells because of its high energy density and simple molecular structure. However, the main focus has shifted towards environmentally friendly materials. Methanol is therefore not applicable as a fuel because of its high toxicity [4]. Additionally, as the fuel supplied to the anode, methanol shows the limitations of inefficient oxidation, low open-circuit potential, and crossover from the anode to the cathode [5]. Therefore, to avoid the problems of methanol, glycerol has become a promising candidate for use in fuel cells. The abundance of glycerol, which is a major product of biodiesel, and its high energy density and low toxicity make this alcohol a good alternative for fuel cell applications [6].

The complex molecular structure of glycerol and the numerous intermediate species in the oxidation process are the primary barriers preventing the use of glycerol in fuel cells. Therefore, the choice of catalyst and reaction conditions is important to ensure the desired outcome. An alkaline medium, rather than an acidic medium, has been used for glycerol oxidation to overcome kinetic constraints during the oxidation reaction [7]. At the anode, the catalyst provides a foundation to convert the chemical energy of the fuel into electrical energy. Since palladium-based materials are efficient anodic materials in alkaline media, bimetallic PdAu nanoparticles supported on vapor-grown carbon nanofibres (VGCNF) are used as catalysts for glycerol oxidation in this study. The properties of PdAu nanoparticles themselves, which have a high tendency to agglomerate, make the use of a catalyst support very important for improving the performance, utilization, and lifetime of the catalyst [8]. Furthermore, in addition to their mechanical strength and surface area in the range of 10–200 m^2^ g^−1^, VGCNF have a unique structure with large number of edges in the lattice and basal regions, which provides a surface for metal-support interactions [9, 10]. The presence of VGCNF as a support material may improve both the dispersion of the metal catalyst and the electrocatalytic performance [11].

The dependence of the electro-oxidation of alcohol on the electrolyte temperature and NaOH concentration has been investigated in several studies. Tripković, Štrbac, and Popović [10] noted that increasing the temperature from 295 to 333 K increased the MOR activity of Pt and PtRu catalysts. Habibi and Razmi [12] studied the effect of NaOH concentration in the range of 0.5 M to 6.0 M and temperature in the range of 25 °C to 80 °C for prepared Au, Pd and Pt nanoparticles supported on a modified carbon ceramic electrode (CCE). The authors reported that the NaOH concentration and temperature directly influenced the oxidation of glycerol. The catalyst loading also affects the alcohol oxidation performance. Basically, reducing the effect of catalyst loading on alcohol oxidation, especially for complex molecules such as glycerol, is a significant challenge. Many studies have [13] developed 10 wt.% to 20 wt.% Pd/C and PdAu/C metal catalyst for the oxidation of ethanol and glycerol. The complexity of polyalcohols, such as ethanol and glycerol that involve many intermediate reaction mechanisms during oxidation, makes it difficult to use lower catalyst loadings.

These observations inspired this optimization study on the reaction conditions of glycerol oxidation. The effects of electrolyte temperature, NaOH concentration and catalyst loading on the performance of glycerol oxidation using PdAu/VGCNF were analyzed by response surface methodology (RSM). As a result, a predictive model was generated from the experimental data by varying one parameter at a time. RSM is an applied statistics technique for experimental design that is used to strategically plan and execute experiments and thereby reduce the number of experiments required to optimize the operational conditions in glycerol oxidation. RSM is a collection of mathematical and statistical techniques based on the fit of a polynomial equation to the experimental data [14, 15]. The use of RSM is more practical because it may include interactive effects among variables and will eventually depict the overall effects that the parameters have on the process [16]. Very limited research has been performed on the operational conditions of the alloyed electrocatalyst. In addition, the RSM optimization of the half-cell performance for glycerol oxidation in alkaline medium using the PdAu/VGCNF catalyst has never been studied. Most studies have focused on the performance of a single cell. However, optimization of the parameters in a half-cell test may provide a benchmark that could be applied to single-cell operation.

## Experimental

### Materials and chemicals

All precursor metal salts and chemical reagents, such as gold(III) chloride trihydrate (HAuCl_4_·3H_2_O), palladium chloride (PdCl_2_), trisodium citrate (Na_3_Ct), sodium borohydride (NaBH_4_), carbon nanofibres, sodium hydroxide, glycerine, 2-propanol, and 5 wt.% Nafion solution, were purchased from Sigma-Aldrich/USA.

### Instrumentation

For the physical analysis of the electrocatalysts, techniques such as X-ray diffraction (XRD), field emission scanning electron microscopy (FESEM), energy dispersive X-ray (EDX) spectroscopy and transmission electron microscopy (TEM) were used to examine the electrocatalyst crystallization, structure, morphology, elemental composition, size, and atomic distribution. XRD is used to identify the phase of crystalline materials. The instrument used in this work is a Bruker D8 Advance diffractometer equipped with a CuKα radiation source at 40 kV and 40 mA. Scanning of the electrocatalyst is performed at a rate of 2° min^−1^ between 30° and 90°. The Scherrer equation is used to determine the size of the crystalline particles in the powder. Topographical and elemental information for the nanostructured catalyst was obtained using a Gemini SEM 500 field emission scanning electron microscope equipped with an energy-dispersive X-ray spectroscope that can provide three-dimensional images as well as provide information on the elemental composition of the sample under analysis. Transmission electron microscopy (TEM) was performed with a Philips CM12 microscope operated at 120 kV. The sample catalyst was placed in ethanol in an ultrasonic bath for 30 min before analysis.

### Catalyst synthesis

The methodological approach to synthesize the electrocatalyst used in this study is a mixed technique based on reduction and impregnation [17]. This is the simplest method that allows the formation of a PdAu bimetallic alloy supported on vapor grown carbon nanofibres (VGCNF). The electrocatalyst synthesis started with 2 ml PdCl_2_ (0.05 M) mixed with 7 ml gold(III) chloride trihydrate (HAuCl_4_·3H_2_O) (0.012 M). The mixed solution was added dropwise to a certain amount of trisodium citrate (0.5 M). Trisodium citrate acts as a stabilizing agent to control the aggregation of the nanoparticles by lowering the surface tension between the solid particles and the solvent. Subsequently, the mixed solution was added dropwise to a stirred VGCNF slurry (isopropanol + DI water) and stirred for 3 h. Reduction of the metal precursors was carried out using an excess amount of freshly prepared ice-cold (0.5 M) sodium borohydride (NaBH_4_), and the solution was stirred overnight. A longer reaction time allows sodium borohydride, with its strong reducing ability, to react with the products. The molar ratio of NaBH_4_ to metal ions is 5 to 15, which provides a better catalyst dispersion and surface composition of the PdAu bimetallic alloy nanoparticles. The solution was kept under magnetic stirring overnight, filtered, washed with DI water several times to remove all of the solvent and dried at 80 °C for 10 h. In the preparation of the electrocatalyst PdAu bimetallic alloy supported on VGCNF, the metal loading was varied between 10 wt.% and 30 wt.%.

### Cyclic voltammetry tests

Cyclic voltammetry experiments were performed for the electrochemical analysis of the electrocatalyst. Cyclic voltammetry measurements were performed using an Autolab (PGSTAT101) electrochemical workstation at room temperature. The catalyst ink was prepared by dissolving 5 mg of electrocatalyst in a mixture of distilled water, isopropyl alcohol, and 5 wt.% Nafion®. A 2.5 μl aliquot of the electrocatalyst ink was deposited on a glassy carbon electrode using a micropipette and then left to dry at room temperature. The electrochemical characterization of the electrocatalysts was performed by a cyclic voltammetry (CV) test over the potential range of − 0.8 to 0.4 V in 1 M NaOH at a scan rate of 50 mVs^− 1^ in 0.5 M glycerol/0.5 M NaOH solution. The concentration and temperature of the NaOH electrolyte varied from 0.5 to 6.0 M and from 25 to 80 °C, respectively. Both solutions were de-oxygenated by bubbling with N_2_ at 200 ml min^− 1^ for 30 min before taking any measurement of the glycerol oxidation reaction.

### Experimental design

Central composite design (CCD) using Design Expert 8.0 was performed to determine the optimization factors for the glycerol oxidation reaction using the PdAu/VGCNF electrocatalyst. CCD is a design tool for sequential experimentation that permits a reasonable volume of information to be tested for a lack of fit when a sufficient number of experimental data points exist [18]. The three factors and ranges used in this work are presented in Table 1 and include the NaOH electrolyte concentration, the electrolyte temperature and the metal loading. The response was set as the current density at the glycerol oxidation peak potential obtained from cyclic voltammetry analysis.

**Table 1:** Factors and level for response surface study

Factor	Low level (−1)	High level(+1)
X_1_:NaOH concentration, M	0.50	6.00
X_2_: Temperature,(°C)	25	80
X_3_: Metal catalyst loading (%)	10	30

The catalyst was prepared for optimization using a combination design matrix, as listed in Table 2, with total of 20 experiments performed, including factorial, axial, and central points. The experimental data were fit to a second-order polynomial regression model, expressed by Eq. 1:

1 $$ Y=\beta o+{\sum}_{i=1}^n\beta i\times Xi+{\sum}_{i=1}^n\beta i i\times X{i}^2+{\sum}_{i=1}^n{\sum}_{j>1}^n\beta i j\times Xi Xj $$

where *Y* is the predicted response variable; *n* is the number of variables; and *β*_0_, *β*_i_, *β*_ii_, and *β*_ij_ are the coefficients of the linear parameters, the quadratic parameters and the interaction parameters, respectively. The certainty of the above polynomial model can be estimated by the coefficient of determination, *R* ^2^. The experiment sequence was randomized to avoid systematic errors.

**Table 2:** Experimental matrix of central composite design runs using Design Expert 8.0 software

Run order no.	Factors variables			Current density (mA cm^−2^)
	X_1_:NaOH concentration (M)	X_2_: Temperature (°C)	X_3_: Catalyst loading	Actual
1	0.5	80	30	115.89
2	3.25	6.25	20	35.89
3	3.25	52.5	20	160.7
4	0.1	52.5	20	113
5	6	25	10	75.62
6	3.25	52.5	6.25	32.33
7	3.25	98.74	20	105.05
8	3.25	52.5	20	150.98
9	0.5	25	10	38.62
10	6	25	30	90
11	6	80	10	101.1
12	3.25	52.5	36	68.98
13	0.5	80	10	110.65
14	6	80	30	119.56
15	3.25	52.5	20	159.7
16	3.25	52.5	20	152.69
17	7.87	52.5	20	164
18	3.25	52.5	20	158.9
19	0.5	25	30	60
20	3.25	52.5	20	163.25

## Results and discussion

### Physical characterization of the catalyst

**Fig. 1:**
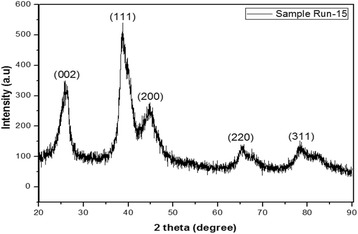
XRD pattern for sample Run-15

To verify the formation of the PdAu alloy supported on VGCNF, a selected sample (sample Run-15) was analyzed by XRD (see Fig. 1). As seen in Fig. 1, the first diffraction peak, centered at 26.0°, can be assigned to graphite-structured carbon in raw VGCNF, specifically the (002) diffraction planes of hexagonal graphite (JCPDS Card No. 41–1487) [10]. The second peak corresponds to a single face-centered-cubic (fcc) phase, indicating that Pd and Au are highly alloyed to form highly alloyed PdAu bimetallic alloy nanoparticles. The sample exhibits XRD peaks at 39.06°, 45.14°, 66.17°, and 79.60° corresponding to the (111), (200), (220), and (311) planes in the fcc structure. The XRD patterns of the PdAu bimetallic alloy can be indexed to the Fm3m space group and the powder diffraction data of JCPDS Card No. 96-151-0339. The addition of a second metal, i.e., Au, shifts the diffraction peaks to lower values due to the interactions of the second metal with Pd. In addition, the XRD peaks for both samples are shorter and broader due to the small size (nanoscale) materials. The crystallite size was estimated using the Scherrer equation, which showed that the crystallite size is 4.5 nm for the Run-15 sample.

**Fig. 2:**
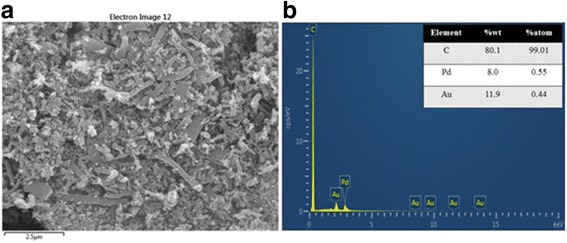
**a** FESEM images and **b** EDX data for sample Run-15

To investigate the morphology of the PdAu particles supported on VGCNF, the sample was observed using FESEM. In Fig. 2a, it can be found that the PdAu particles supported on VGCNF have a moderate degree of agglomeration on VGCNF, and their shape is difficult to distinguish. The elemental composition distribution within the catalyst sample was measured by EDX, given in Fig. 2b. When the ratio of PdCl_2_: HAuCl_4_·3H_2_O in the feed solution was 1:1, the Pd: Au elemental ratios of were determined to be 55:44 which is closed to the expected ratio. This undoubtedly confirmed the presence of Pd and Au nanoparticles and in good agreement with those of the two metal salts in the feed solutions.

**Fig. 3:**
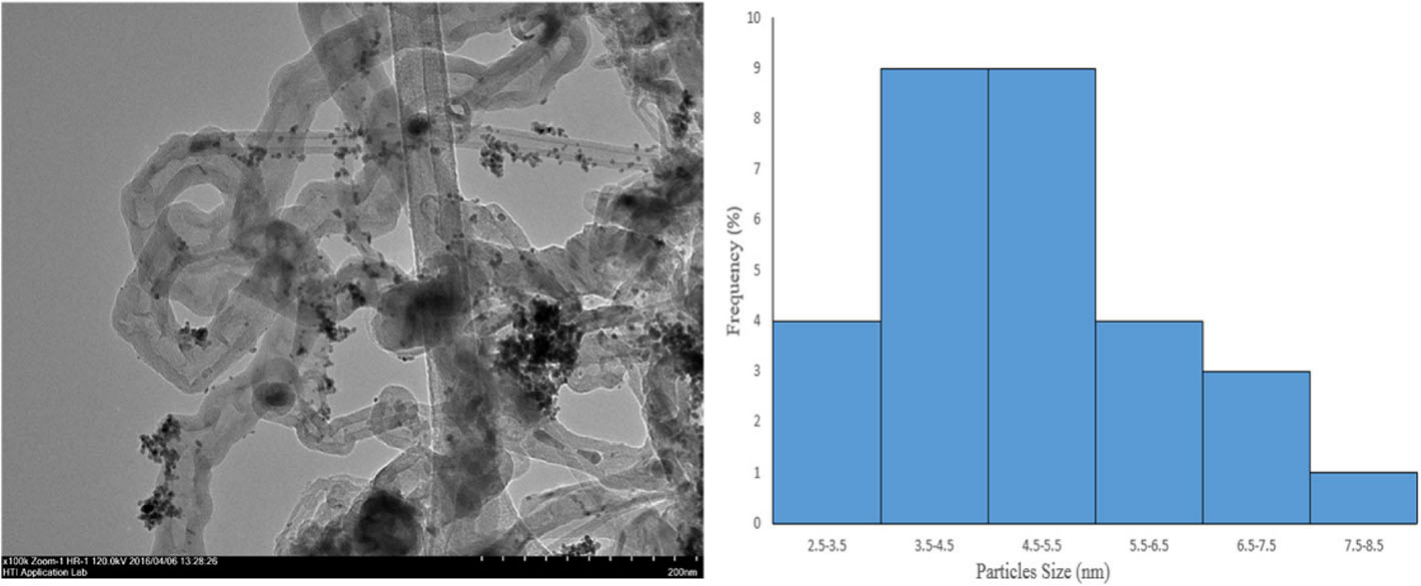
TEM images for sample Run-15

Figure 3 shows the TEM image of PdAu/VGCNF (sample Run-15). The images of the samples show that the PdAu nanoparticles are well distributed on VGCNF, with small particles sizes in the agglomerated and aggregated mixture. The agglomerated particles do not form hard aggregates but soft agglomerates consisting of primary particles weakly attached by van der Waals and capillary adhesive forces [19]. This may be due to long-range magnetic dipole-dipole interactions between the particles. In addition, this result was also observed during the drying step in TEM sample preparation resulting from capillary forces during solvent evaporation [20]. The particle size distribution histogram ranges between 2.5 and 9.5 nm, with an average diameter of 4.5 ± 1.0 nm. These values are close to the crystallite sizes obtained from XRD analysis.

### Optimization study

Table 2 shows the current density response at the peak potential of the glycerol oxidation reaction. The current density at the peak potential of glycerol oxidation is modeled by the second-order polynomial regression given by Eq. 1. The fit summary model statistical results obtained from Eq. 1 are shown in Table 3. Modeling of the second-order polynomial regression is used to maximize the adjusted and predicted R^2^ values. As shown in Table 3, the quadratic model has the highest adjusted *R* ^2^ and predicted *R* ^2^ values and the lowest *p* value.

**Table 3:** Fit summary model statistic result

Source	Std Dev.	*R* ^2^	Adjusted *R* ^2^	Predicted *R* ^2^	*p* value
Linear	43.35	0.2345	0.0910	− 0.1347	0.2211
2F1	47.54	0.2520	− 0.0932	− 0.6137	0.9578
Quadratic	7.45	0.9859	0.9732	0.9089	< 0.0001
Cubic	6.57	0.9934	0.9791	0.1901	0.2643

Table 4 shows the analysis of variance (ANOVA) results for the current density of the glycerol oxidation reaction. The p-value of the model is < 0.0001, which indicates that the model is significant [21]. The factors used for this study, i.e., NaOH concentration, electrolyte temperature and catalyst loading, are all significant in the model of the glycerol oxidation reaction. The model also has a high *R* ^2^ determination coefficient, with a value of 0.9859, indicating that the model fits the observed data well, with only 0.0141% variability in the response. The empirical model is adequate and signifies good model performance if the model has a *R* ^2^ value of at least 0.75 [22]. The p-value for the lack of fit is 0.0844, which is greater than 0.05; this also shows that the model fits well and that there is a significant correlation between the parameters and the output response [23], as shown in Table 4. The gap between the predicted *R* ^2^ and adjusted *R* ^2^ is not more than 0.3, which implies that the non-significant terms do not interfere in the quadratic model. The degree of freedom (*F* test) in the model has a value of 4303.03, which implies that the model is significant and that there is only a 0.01% chance that this large *F* value could occur due to noise. The coded factors model was developed to fit the quadratic model obtained in Eq. 2;

2 $$ \mathrm{Current}\  \mathrm{Density}=157.49+{10.76}^{\ast }{\mathrm{X}}_1+{21.91}^{\ast }{\mathrm{X}}_2+{8.87}^{\ast }{\mathrm{X}}_3-{5.37}^{\ast }{\mathrm{X}}_1{}^2-{29.43}^{\ast }{{\mathrm{X}}_2}^2-{36.43}^{\ast }{{\mathrm{X}}_3}^2-{9.11}^{\ast }{{\mathrm{X}}_1}^{\ast }{\mathrm{X}}_2+{0.78}^{\ast }{{\mathrm{X}}_1}^{\ast }{\mathrm{X}}_3-{1.51}^{\ast }{\mathrm{X}}_2\ast {\mathrm{X}}_3 $$

**Table 4:** ANOVA for current density of oxidation reaction of glycerol

Source	Sum of squares	DOF	Mean square	*F* value	Probability > *F*
Model	38,727.25	9	4303.03	77.52	< 0.0001^a^
X_1_: NaOH concentration	1579.95	1	1579.95	28.46	0.0003
X_2_: Temperature	6558.19	1	6558.19	118.14	< 0.0001
X_3_: Catalyst Loading	1073.79	1	1073.79	19.34	0.0013
X_1_^2^	416.27	1	416.27	7.50	0.0209
X_2_^2^	12,479.20	1	12,479.20	224.80	< 0.0001
X_3_^2^	19,128.37	1	19,128.37	344.59	< 0.0001
X_1_X_2_	663.94	1	663.94	11.96	0.0061
X_1_X_3_	4.84	1	4.84	0.087	0.7739
X_2_X_3_	18.18	1	18.18	0.33	0.5798
Residual	555.11	10	55.51		
Lack of fit	439.61	5	87.92	3.81	0.0844^b^
Pure Error	115.50	5	23.10		
Cor Total	39,282.36	19			
*R* ^2^	0.9859				
Adj *R* ^2^	0.9732				
Pred *R* ^2^	0.9089				

^a^Significant, ^b^Not significant

**Fig. 4:**
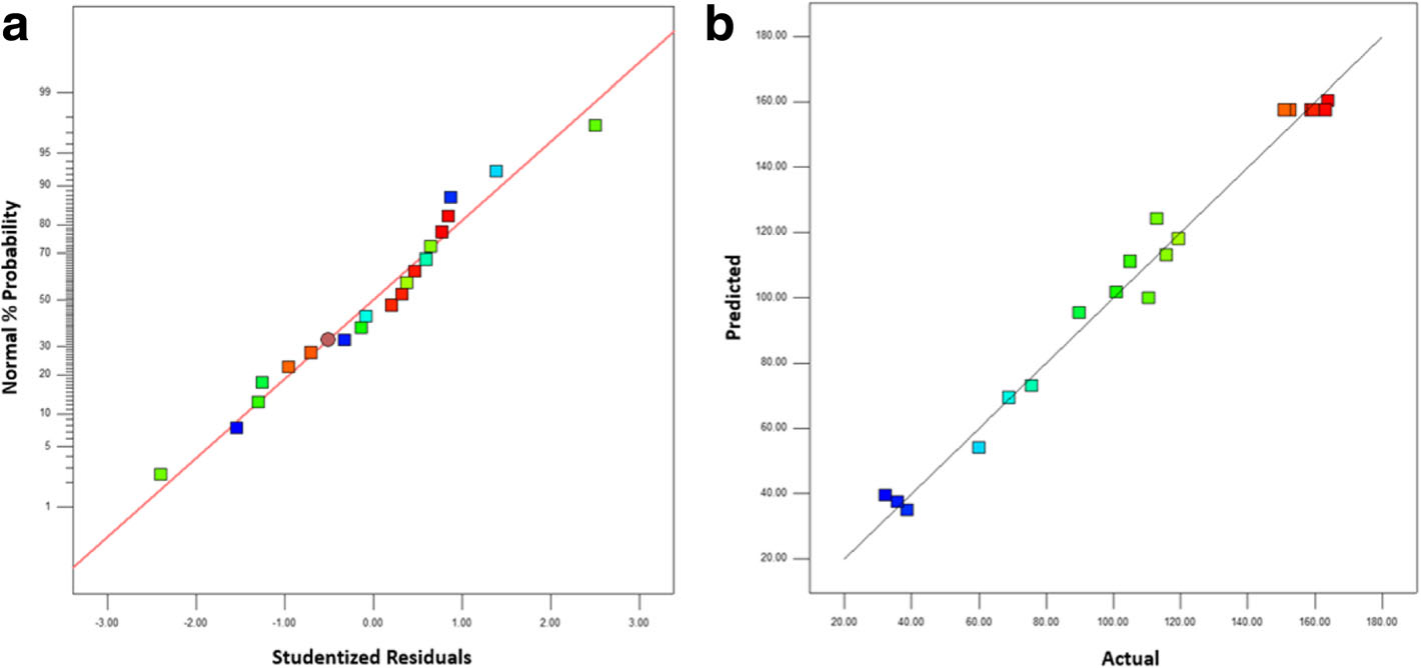
**a** Normal probability plot of the studentized residuals in response surface methodology (RSM). **b** The actual response data vs. the predicted data for the current density at the oxidation peak of the glycerol oxidation reaction

**Fig. 5:**
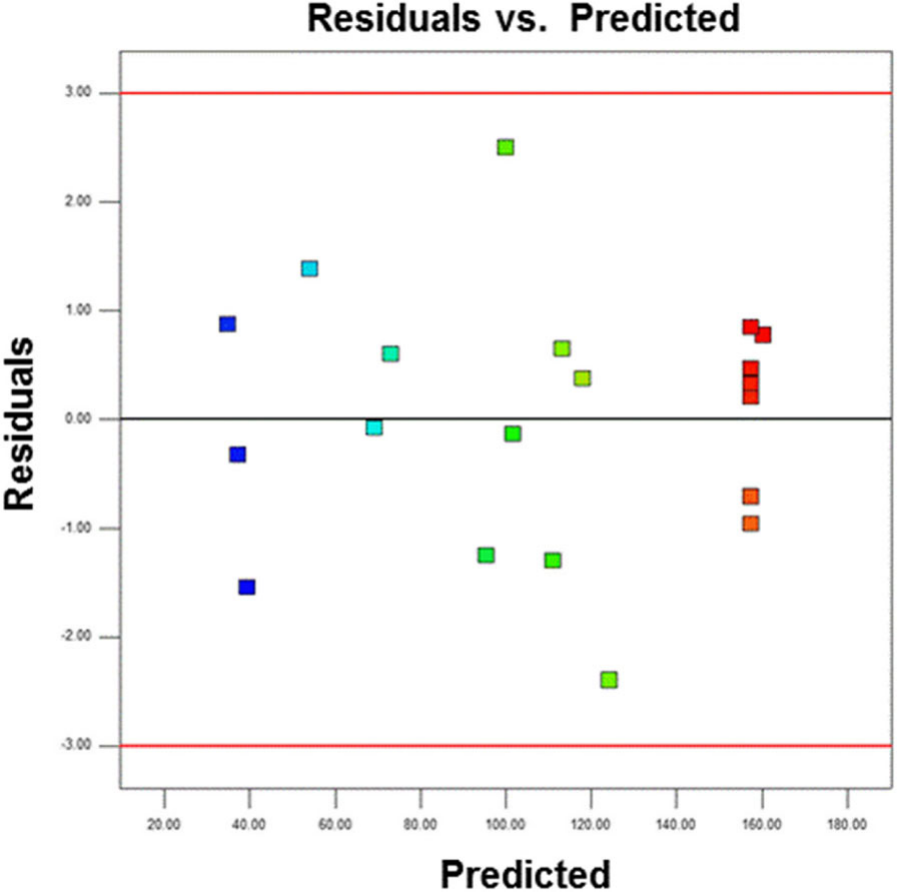
Plot of residuals vs. prediction for the raw data

Figure 4a shows the normal probability plot of the studentized residuals. The plot shows that the data points are approximately linear, indicating the desired normality in the error terms. Figure 4b shows the plot of the actual response data versus the predicted current density at the oxidation peak of the glycerol oxidation reaction. The plot of the predicted versus experimental current density (mAcm^−2^), which fits the regression model perfectly, is in good agreement with the observed density in the range of the operating variables. Figure 5 shows the plot of residuals vs. predicted values for the raw data. This plot is used to check the adequacy of the model. In Fig. 5, the plot of the standardized residuals versus the run order shows that the residuals are scattered randomly along a straight line. This result suggests that the variance of the original observations is constant for all response values.

### Performance of glycerol oxidation under different conditions of interactive parameters

**Fig. 6:**
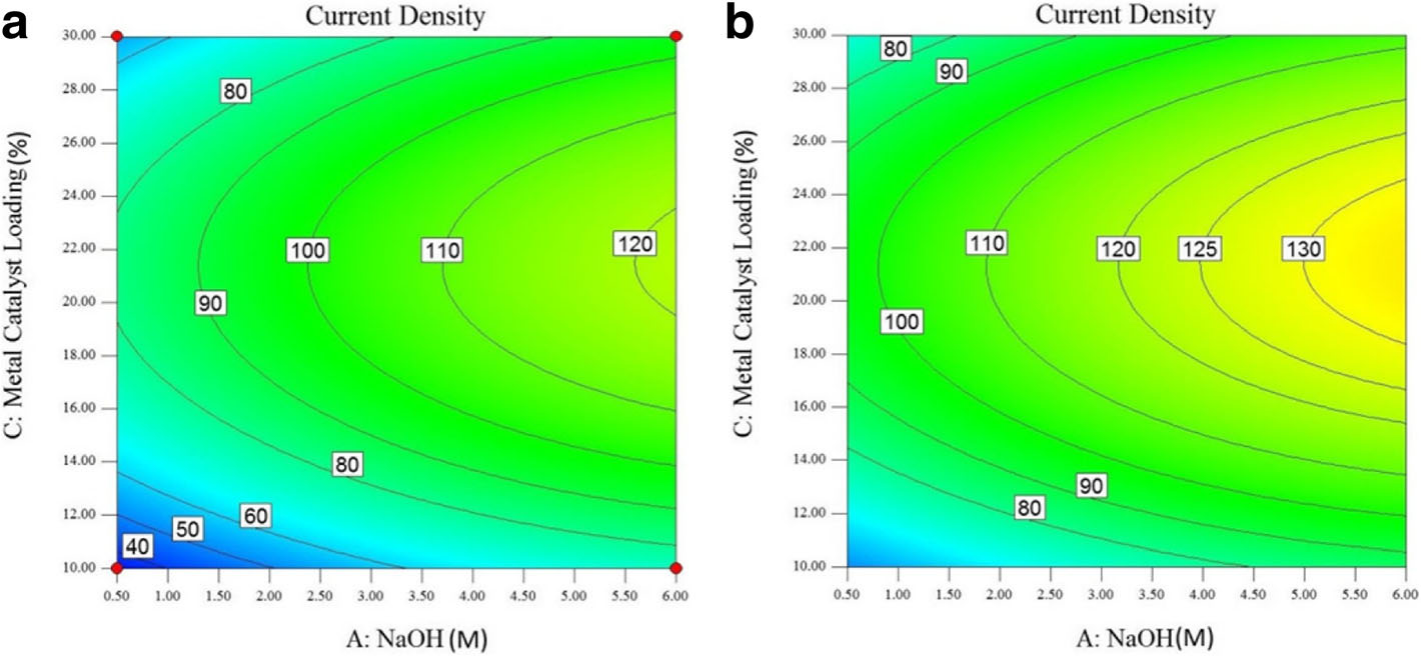
Plot of the current density at the oxidation peak of the glycerol oxidation reaction as a function of metal catalyst loading (wt.%) and NaOH concentration (M) at electrolyte temperatures of (**a**) 25 °C and (**b**) 30 °C

**Fig. 7:**
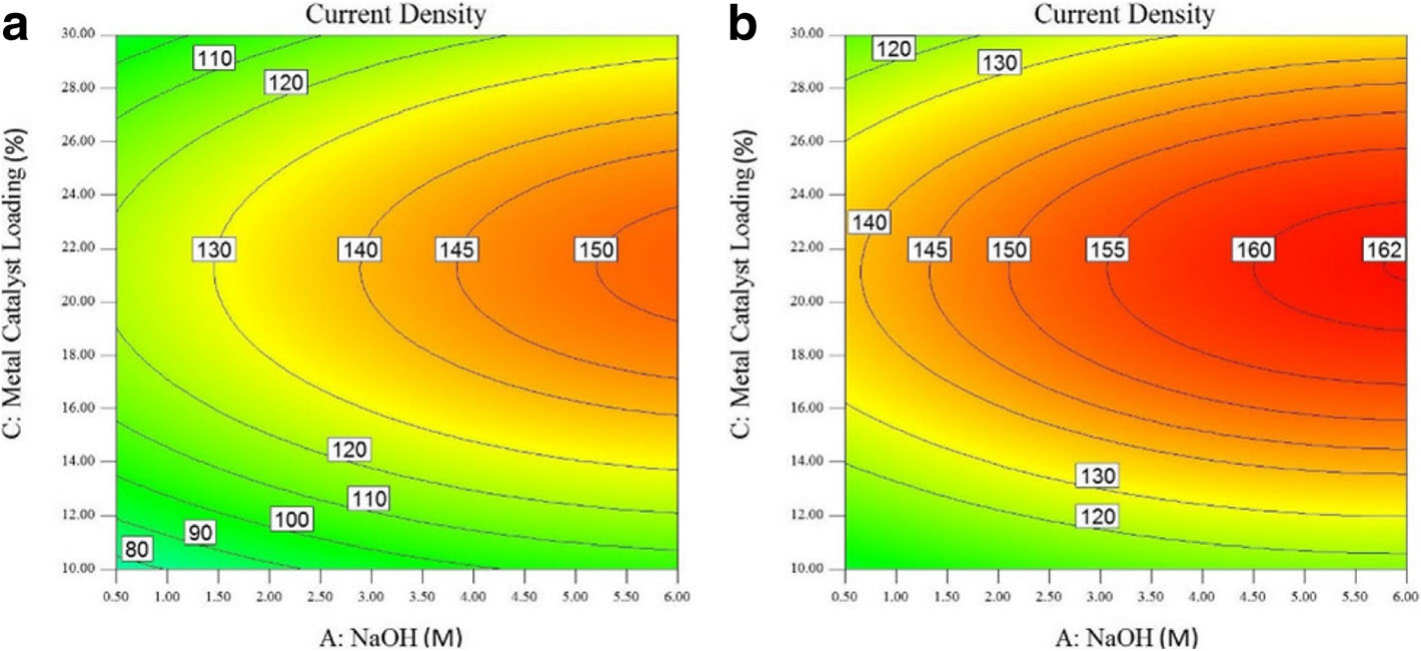
Plot of the current density at the oxidation peak of the glycerol oxidation reaction as a function of metal catalyst loading (wt.%) and NaOH concentration (M) at electrolyte temperatures of (**a**) 40 °C and (**b**) 50 °C

**Fig. 8:**
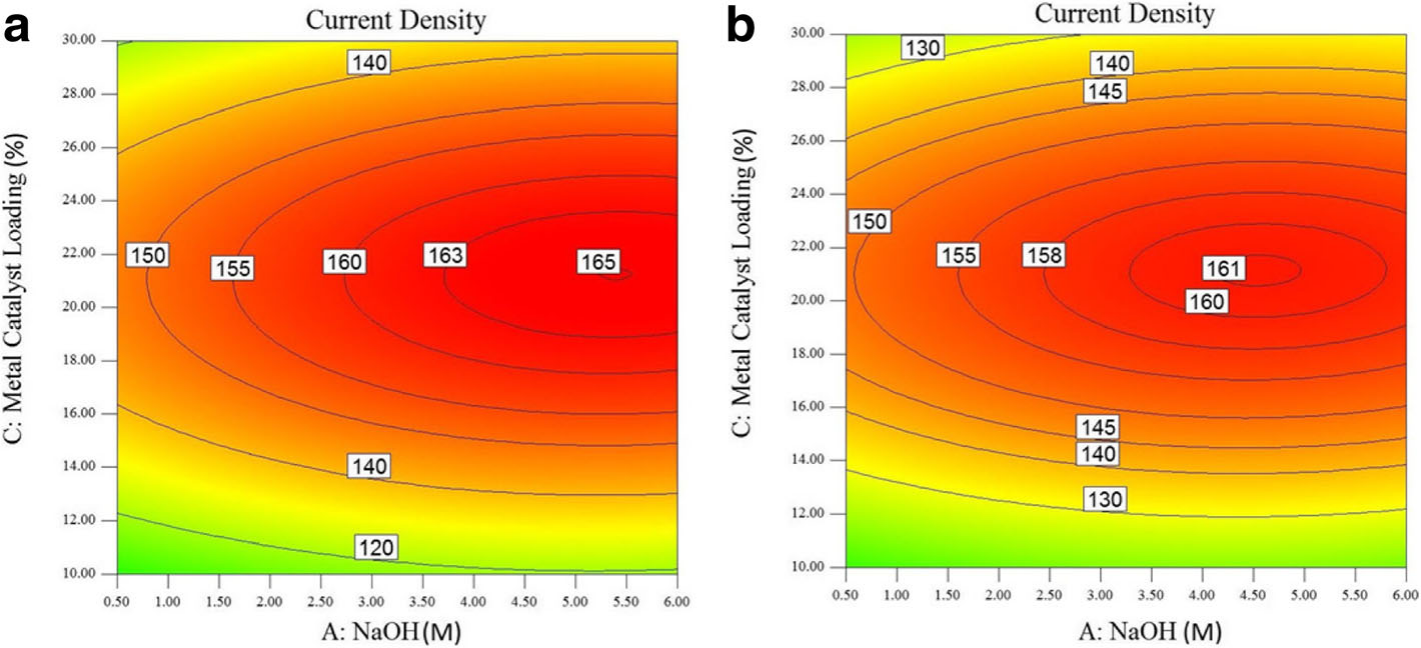
Plot of the current density at the oxidation peak of the glycerol oxidation reaction as a function of metal catalyst loading (wt.%) and NaOH concentration (M) at electrolyte temperatures of (**a**) 60 °C and (**b**) 70 °C

Figure 6, 7, 8, and 9 show contour plots of the current density at the oxidation peak of glycerol oxidation by the PdAu/VGCNF catalyst as a function of the metal catalyst loading (wt.%) and NaOH electrolyte concentration (M) at different electrolyte temperatures ranging from 25 to 80 °C. Figure 6a shows the contour plot when the electrolyte temperature is set at 25 °C. As seen in Fig. 6a, the current density increases slightly as the NaOH concentration and metal catalyst loading increase. However, at a metal catalyst loading of over 22 wt.%, the current density decreases. The contour plot shows that the highest current density attained at 25 °C is 120 mAcm^−2^. At this current density, a metal catalyst loading of 18–24 wt.% and NaOH concentration of 5.5–6.0 M are required. The same pattern of the contour plots of the current density at the oxidation peak of the glycerol oxidation reaction at 30 °C is observed in Fig. 6b. The current density tends to decrease at over 24 wt.% metal loading. The region of high current density occurs when 130 mAcm^−2^. This area requires a NaOH concentration of 5.0–6.0 M and a metal catalyst loading between 18 wt.% and 24 wt.%. Similar high current densities can be reached at both 25 and 30 °C, but the NaOH concentration must decrease to 5.0 M to obtain a high current density for the oxidation peak of the glycerol oxidation reaction.

**Fig. 9:**
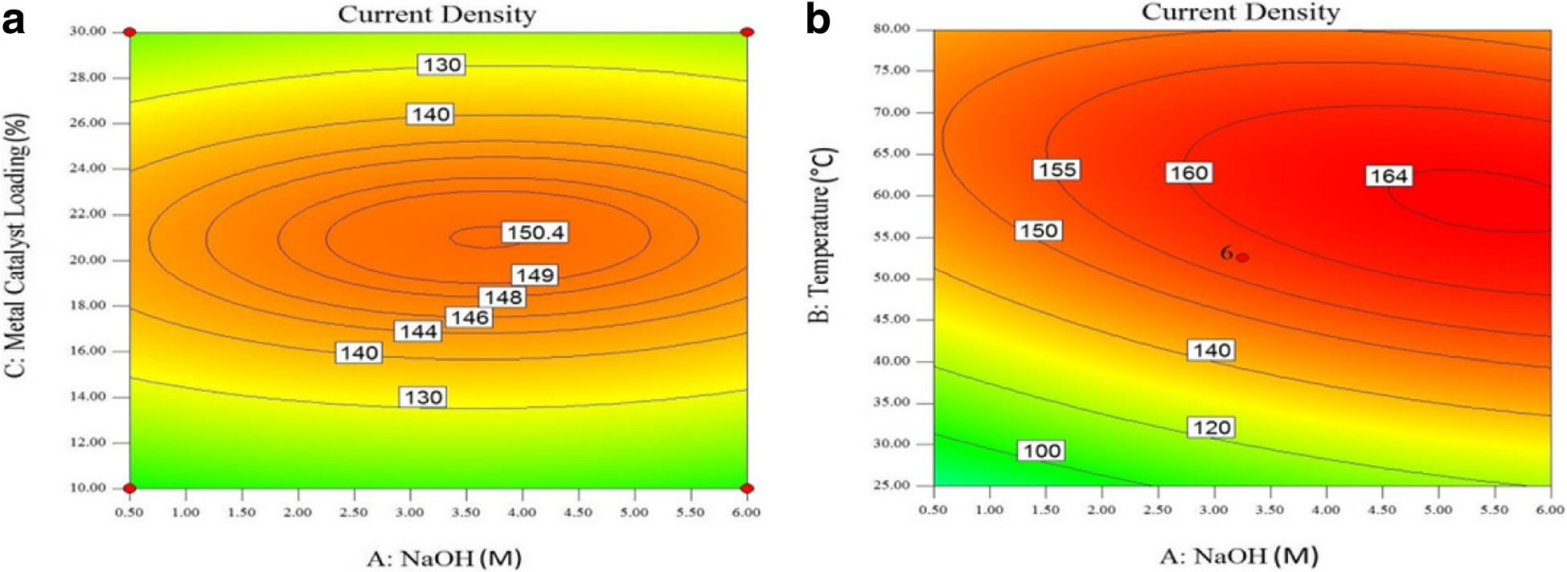
Plot of the current density at the oxidation peak of the glycerol oxidation reaction (**a**) as a function of metal catalyst loading (wt.%) and NaOH concentration (M) at an electrolyte temperature of 80 °C and (**b**) as a function of electrolyte temperature (°C) and NaOH concentration (M) at 20 wt.% metal catalyst loading

Figure 7a shows the contour plot of the current density of the oxidation peak of the glycerol oxidation reaction at an electrolyte temperature of 40 °C. The highest current density that can be attained at this temperature is 150 mA/cm^2^, in contrast to the 130 mA/cm^2^ attained at 30 °C. Compared with the 30 °C electrolyte temperature, the metal loading can be in the range of 16–29 wt.% with a NaOH concentration ranging from 1.50 to 6.0 M to obtain a 130 mA/cm^2^ current density with an electrolyte temperature of 40 °C. However, using a NaOH concentration ranging from 5.0 to 6.0 M with a metal catalyst loading reduced by 2 wt.% (20–24 wt.%) would achieve the highest current density of 150 mA/cm^2^ at a 40 °C electrolyte temperature; a temperature of 30 °C can achieve a current density of only 130 mA/cm^2^. The change of the electrolyte temperature from 30 to 40 °C increases the current density at the oxidation peak of the glycerol oxidation reaction.

The contour plot of the current density of the oxidation peak of the glycerol oxidation reaction when the electrolyte temperature is further increased to 50 °C is shown in Fig. 7b. The highest current density at this temperature is 162 mA/cm^2^, but the area is small, and a metal catalyst loading and NaOH concentration of 21–22 wt.% and 5.75–6.0 M, respectively, are required. At an electrolyte temperature of 50 °C, using the same range of metal catalyst loading (20–24 wt.%) shifts the NaOH concentration by 0.5 M (4.5–6.0 M) to obtain the current density of 160 mA/cm^2^. The effect of temperature increases the current density to a high value for the same range of metal catalyst loading and NaOH concentration.

Figures 8a, b and 9a show the contour plots of the current density at the oxidation peak of the glycerol oxidation reaction at 60, 70, and 80 °C, respectively. In Fig. 8a, the current density has the highest value of 165 mAcm^− 2^ at 60 °C compared to that at 70 and 80 °C, which show current densities of 161 mAcm^− 2^ and 150.4 mAcm^− 3^, respectively. The PdAu/VGCNF catalyst obtains the highest current density at the oxidation peak of the glycerol oxidation reaction at 60 °C. At temperatures greater than 60 °C, the current density decreases. In Fig. 8a, the NaOH concentration required to obtain the highest current density is in the range of 5.0–5.5 M. However, obtaining a current density of 160 mA/cm^2^ requires a NaOH concentration as low as 3 M, which is the lowest concentration found in this study. The highest current density at an electrolyte temperature of 70 °C decreases to 161 mA/cm^2^, and the NaOH concentration ranges from approximately 4.0–5.0 M. Increasing the temperature to as high as 80 °C reduces the highest current density to 150.4 mA/cm^2^ as well as the NaOH concentration to the 3.5–4.0 M range.

The metal catalyst loadings needed to obtain the highest current density at temperatures ranging from 60 to 80 °C seem to be same, approximately 20–24 wt.%. Increasing the metal loading further only reduces the current density. The same conditions are also applied to other temperatures. Increasing the metal catalyst loading to over 24 wt.% may block the active sites for the glycerol oxidation reaction. The catalyst is active and allows the adsorption of glycerol onto the surface of the catalyst. However, the amount of catalytic metal on the support must be considered. A high catalyst loading will affect the thickness of the fuel cell catalyst layer due to the large volume of the carbon support. Furthermore, increasing the metal loading can contribute to the saturation of the electrochemically active surface area (EASA) [24]. This may be due to the high likelihood of Pd aggregation, even in the presence of the support. Therefore, a high metal loading will increase the degree of nanoparticle aggregation and reduce the porosity, which may result in mass transport limitations and reduce the catalytic activity [25]. If the temperature and catalyst loading are increased simultaneously, the decrease in the current density may cause the PdAu alloy particles to cluster, leading to limited mass activity because of the very rapid reaction rate of the redox trans-metalation reaction for the PdAu catalyst [26]. Figure 9b shows the current density at the oxidation peak of the glycerol oxidation reaction with a metal catalyst loading of 20 wt.% as a function of electrolyte temperature and NaOH concentration. By setting the metal catalyst loading constant at 20 wt.%, the electrolyte temperature and NaOH concentration can be varied to obtain the optimum current density.

The increase in the current density results from the temperature of the electrolyte due to the improvement of the diffusion coefficients, mass transfer of the reactants and reaction kinetics. The glycerol molecules move faster when heat is introduced, thus enabling faster glycerol transport to the anode catalyst. However, increasing the temperature to above 65 °C did not have a significant effect on the current density; to be more precise, the current density became stagnant due to the formation of intermediate species, which might block the active sites and decay the performance of the catalyst [27]. This is also observed for increased NaOH concentrations with a constant electrolyte temperature. The current density increases to 123.33 mAcm^− 2^ at a NaOH concentration of 6.0 M and a temperature of 25 °C, as shown in Fig. 9b. The current density increases because the increased OH^−^ concentration in an alkaline electrolyte environment may give rise to greater OH^−^ coverage on the catalyst surface. The presence of OH^−^ facilitates the adsorption of glycerol on the catalyst active sites, and increasing the OH^−^ concentration to a certain value will prevent the adsorption of glycerol on the catalyst sites and decrease the reaction rate of the glycerol oxidation [28]. Figure 9b shows the decrease in the current density when the temperature and NaOH concentration approach 80 °C and 6.0 M, respectively. In general, the performance of the catalyst increases with increasing temperature and electrolyte concentration. However, at a certain point, these two operating conditions will have an adverse effect on the current density. Temperatures and NaOH concentrations that are too high will lead to a higher coverage of the active layer on the anode catalyst and a decrease in the cell performance [27]. The highest current density is 164 mAcm^− 2^, recorded at a NaOH concentration of 6.0 M and a temperature of 60 °C.

### Confirmation test

**Fig. 10:**
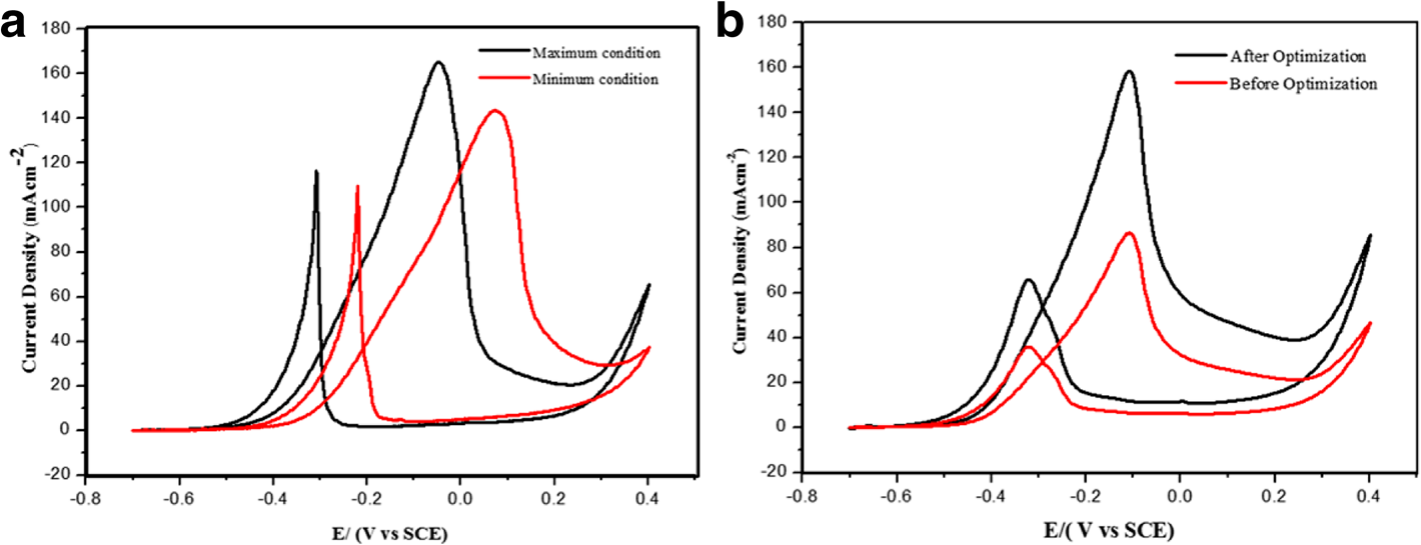
Plot of the current density for (**a**) the maximum and minimum conditions and (**b**) before and after optimization of the reaction conditions

Two additional confirmation experiments were conducted to validate the RSM model and ensure that the model provides an adequate approximation to the real system. The chosen conditions for the temperature, NaOH concentration and catalyst loading, together with the predicted and experimental results, are listed in Table 5. Figure 10 shows the experiments that were performed to verify the accuracy of the developed model. The predicted and experimental values were compared, and the margin of the error was in the permissible range. The maximum current density of 164.10 mAcm^− 2^ was recorded during the cyclic voltammetry test at a NaOH concentration of 6.0 M, temperature of 50 °C and catalyst loading of 20 wt.%. These conditions affected the glycerol oxidation performance of the catalyst, producing the best current density. For the second set of conditions, the NaOH concentration and temperature were set to the minimum values, with a NaOH concentration of 0.5 M, temperature of 45.21 °C and catalyst loading of 20 wt.%. The maximum current density achieved in the experiment was 143.94 mAcm^− 2^. Although the current density was slightly lower, the system can be run with minimal operational cost. In addition, reducing the temperature reduces the heat or energy of the system. Reducing the energy usage directly decreases the operational cost. One optimal condition, NaOH concentration of 5.24 M, temperature of 60 °C and minimal catalyst loading of 12 wt.%, was found that led to a current density of 158.34 mAcm^− 2^ during glycerol oxidation. Compared to the conditions used before the optimization, the catalyst loading can be minimized by up to 8%, and the current density can be increased by more than 40% (Fig. 10). The parameters chosen for the optimum conditions are suitable for single-cell operation. Table 6 presents a comparison of our Pd-based catalyst with that used in a previous study and shows that the oxidation of glycerol is remarkably enhanced when using the PdAu/VGCNF catalyst after optimizing several of the reaction parameters.

**Table 5:** Experimental and predicted current density in two different condition a) Optimum parameters for maximum current density. b) Maximum current density in minimum operational cost. c) Current Density before optimization and d) current density after optimization

Run	NaOH concentration, M	Temperature, °C	Metal catalyst loading, (wt%)	Predicted current density, mAcm^−2^	Experimental current density, mAcm^−2^	Condition
a	6.00	50	20	161.41	164.10	Maximum
b	0.50	45	20	130.98	143.94	Minimum
c	1.00	25	20	–	86.36	Bef. Optimization
d	5.24	60	12	150.02	158.34	Aft. Optimization

**Table 6:** Performance comparison with different electrocatalysts base on Pd in GOR in alkaline media with the scan rate of 50 mVs^−1^

Catalyst	Fuel	Metal catalyst loading (wt%)	Concentration NaOH (M)	Temp (°C)	Current density (mAcm^−2^)	References
Pt/CCE Pd/CCE Au/CCE	Glycerol (0.5 M)	20 wt%	0.01–4.0 M	25–80	46.8 mAcm^−2^ 51.8 mAcm^−2^ 58.0 mAcm-2	[9]
Pd_m_Au/C, Au/C and Pd/C	Ethanol (0.5 M)	20 wt%	2.0 M	25	10.6 mA μg^−1^	[14]
Pd–Au	Methanol (1.0 M)	60 wt%	0.5 M	25	1.30 mA cm^−2^	[27]
Pd/C, PdAu/C 50:50, PdSn/C 50:50, PdAuSn/C 50:40:10 and PdAuSn/C 50:10:40	Glycerol (1.0 M)	–	1.0 M	25	70 mAcm^−2^	[10]
PdAg (1:1) /C PdAg (3:1) / C PdAu (1:1)/C PdAu (3:1)/C	Glycerol (0.5 M)	–	1.0 M	25	13.83 mA/cm^2^	[28]
Pd-Au NRs	Ethanol (1.0 M)	–	0.1 M	25	26.58 mAcm^−2^	[29]
PdAu /VGCNF	Glycerol (0.5 M)	20 wt%	1.0 M	25	86.26 mAcm^−2^	Before Optimization
PdAu /VGCNF	Glycerol (0.5 M)	12 wt%	5.24 M	60	158.34 mAcm^−2^	After Optimization

## Conclusions

Response surface methodology using central composite design is a powerful method for the examination and optimization of multivariable procedures. In this study, the Design Expert RSM tool generated 20 experiments to analyze the effects of temperature, NaOH concentration and catalyst loading on the current density of the glycerol oxidation reaction via cyclic voltammogram testing. According to the *F* values in the analysis of variance (ANOVA) evaluation, the NaOH concentration and temperature of the electrolyte had significant effects on the response. High temperatures improved the reaction kinetics of the glycerol reaction. Meanwhile, a high NaOH concentration provided OH^−^ ions that facilitated the glycerol oxidation reaction. The best expression or optimal conditions subject to the highest current density of 158.34 mAcm^− 2^ were found to be at a NaOH concentration, temperature and catalyst loading of 5.24 M, 60 °C and 12 wt.%, respectively. In conclusion, using RSM to optimize an analytical method verified and successfully determined the optimum conditions for glycerol oxidation when using PdAu/VGCNF as the catalyst.
